# Exploring the exercise intensity equivalent to the anaerobic threshold in patients with acute myocardial infarction based on the 6-minute walk test distance

**DOI:** 10.3389/fcvm.2025.1452802

**Published:** 2025-02-25

**Authors:** Yuxuan Fan, Xiaopeng Sun, Guihua Li, Xiaojing Wang, Qian Luo, Lemin Wang, Yuqin Shen, Zhiqing Fan

**Affiliations:** ^1^ Department of Rehabilitation, Tongji Hospital Affiliated to Tongji University, Tongji University School of Medicine, Shanghai, China; ^2^ Department of Cardiology Rehabilitation, Daqing Oilfield General Hospital, Daqing, Heilongjiang, China; ^3^ Department of Cardiology, The Second Affiliated Hospital of Dalian Medical University, Dalian, China

**Keywords:** exercise rehabilitation, acute myocardial infarction, 6-minute walk test, anaerobic threshold, exercise intensity

## Abstract

**Objective:**

This study aimed to evaluate the correlation between aerobic exercise intensity based on the 6 min walk test (6MWT) and the anaerobic threshold (AT)-based equivalent in patients with acute myocardial infarction (AMI). The feasibility of using the 6MWT for exercise prescription in primary care settings was also investigated.

**Methods:**

A retrospective analysis was conducted on data from AMI patients, including statistics on all values of the cardiopulmonary exercise test and 6MWT parameters.

**Results:**

Regression analysis showed that the regression equation based on 6MWD exercise intensity (EI_6MWD_) could predict AT-based exercise intensity (EI_AT_). Moreover, EI_6MWD_ correlated with EI_AT_ in 91.9%–93.0% of patients' EI_6MWD_, with AMI equivalent to the EI_AT_ model.

**Conclusions:**

The findings suggest that the anaerobic threshold in AMI patients corresponds to 91.9%–93.0% of the distance covered during the 6MWT. Thus, the 6MWT is a feasible tool for developing exercise prescriptions in primary care hospitals.

## 1 Introduction

Acute myocardial infarction (AMI) is a leading cause of mortality and a major threat to human health (1), with the number of cases rising annually (2). Advances in coronary interventional techniques and clinical management have increased post-surgical survival rates. However, disability and hospitalization rates have also significantly increased (3). Therefore, facilitating early recovery after discharge and reducing readmissions remain critical public health challenges.

Cardiac rehabilitation plays a vital role in improving vascular endothelial and cardiac function, promoting collateral circulation, and preventing the onset and progression of heart failure after AMI. It has been shown to enhance quality of life while reducing the risks of morbidity and mortality associated with cardiovascular disease (4–8). Hence, implementing safe and effective individualized exercise prescriptions as early as possible is essential for optimizing recovery, treatment, and prognosis in AMI patients. The anaerobic threshold (AT) is currently recoginized as a reliable criterion for determining exercise intensity in cardiac rehabilitation for AMI patients (4, 5).

A key factor in effective rehabilitation is selecting an appropriate aerobic exercise intensity for each patient. The cardiopulmonary exercise test (CPET) is considered the gold standard for assessing aerobic capacity with previous studies using it to calculate peak oxygen uptake (peak VO_2_) and AT (9). Nevertheless, the specialized equipment and high costs associated with CPET limit its widespread clinical application. The 6MWT, a submaximal functional capacity test commonly used in cardiac rehabilitation, offers a practical alternative due to its simplicity, low cost, and ease of operation in all hospitals, especially primary hospitals (10–12). However, AT and peak VO_2_ cannot be directly or accurately measured using the 6MWT (13, 14). Consequently, no validated method currently exists to develop an exercise prescription equivalent to AT intensity from 6MWT results for guiding exercise rehabilitation in post-AMI patients.

This study aimed to establish an aerobic exercise intensity equivalent to AT for AMI patients based on the 6 min walk distance (6MWD), facilitating the development of cardiac rehabilitation in primary care settings in China.

## 2 Methods

### 2.1 Study population

The data for this study were obtained from a retrospective observational analysis of AMI patients who completed both CPET and 6MWT between December 2016 and December 2022 at the Department of Cardiac Rehabilitation, Daqing Oilfield General Hospital. Inclusion criteria were: (1) age 18–75 years, (2) stable symptoms and signs of myocardial infarction for over 2 weeks, and (3) within 12 months of AMI surgery (15). Patients were excluded if they (1) did not complete the 6MWT with a cardiac ultrasound within 1 day before or after CPET, (2) had mobility problems, uncontrolled hypertension, severe cardiopulmonary failure, malignant ventricular arrhythmias, severe combined hepatic or renal failure, severe cerebrovascular pathology or psychiatric illness, or severe valvular heart disease or cardiomyopathy, or (3) were more than 12 months postoperative AMI (15, 16). Patients were grouped into ST-segment elevation myocardial infarction (STEMI) and non-ST-segment elevation myocardial infarction (N-STEMI) groups according to their electrocardiogram (ECG) changes. All procedures were conducted by a comprehensive cardiac rehabilitation team consisting of 1–2 cardiologists and 1–2 physical therapists.

This study adhered to the Declaration of Helsinki (as revised in 2013) and was approved by the Daqing Oilfield General Hospital Ethics Committee (ZYAF/SC-07/02.0). Informed consent was obtained from all patients.

### 2.2 6MWT

The 6MWT was performed on a 30-meter flat ground marked at 3-meter intervals. Patients were instructed to walk back and forth along the prescribed test path at their own pace without running. Tests followed uniform standards (17), and the following parameters were recorded: heart rate (HR), peripheral oxygen saturation (SpO2), blood pressure (BP), and symptoms of dyspnea and dizziness, assessed using the Rate of Perceived Exertion (RPE) scale (6–20). The total walk distance was measured, and the average of two test rounds was taken as the final 6MWT result. The intensity of aerobic exercise was determined which based on the patients' average walking speed, termed EI_6MWD_, calculated as: EI_6MWD_ _=_ 6MWD × 10/1,000 (km/h). For instance, a patient covering 350 meters in the 6MWT would achieve an EI_6MWD_ of 3.5 km/h.

### 2.3 CPET

All patients underwent CPET at the Daqing Oilfield Cardiac Rehabilitation Department, following the American College of Cardiology's standard of care and the standard continuous incremental power program used at the Harbor-UCLA Medical Center (4, 18). Testing was conducted using a pulmonary function test system, an exercise test system, and an electric bicycle (CS200, Schiller, Switzerland). The CPET protocol included a 3 min rest period, a 3 min unloaded cycling phase, followed by an incremental workload increase from 0 W/s, with increments of 10–30 W/min based on the patient's age, sex, and estimated functional status. Patients exercised to their symptom-limited maximal effort within 6–10 min, followed by a 5–10 min recovery periode. Patients' resting and peak heart rates, blood pressure, and expiratory breaths were recorded during the CPET. The anaerobic metabolic thresholds were determined using the V-slope method (19). Aerobic exercise intensity, termed EI_AT_, was determined using AT. The value of EI_AT_ was calculated using the following formula based on metabolic equivalents (METs): EI_AT_ = (METs@AT-1) × 3.5 × 60/100 (km/h). The metabolic equivalent (MET) values were determined by the conversion of aerobic exercise oxygen consumption (1 MET = 3.5 ml/kg/min) (20, 21).

### 2.4 Statistical analysis

Normally distributed data are shown as mean ± standard deviation (SD), and Pearson's cumulative correlation was used to analyze relationships. Non-normally distributed measures are presented as medians (*M*) with interquartile spacing (*P*_25_, *P*_75_), and their relationships were analyzed using Spearman's correlation. Count variables are expressed as composition ratios (%). Correlations between unordered variables (sex) and the measurement data were analyzed using the independent samples *t*-test, while associations between unordered categorical variables (sex) were assessed using the chi-square test. Multiple linear regression analysis included variables that were significant in univariate analysis and clinically significant. Data were analyzed using SPSS 22.00, with two-tailed testing, and statistical significance was set at *P* < 0.05.

## 3 Results

### 3.1 Baseline information and clinical data

**Figure 1:**
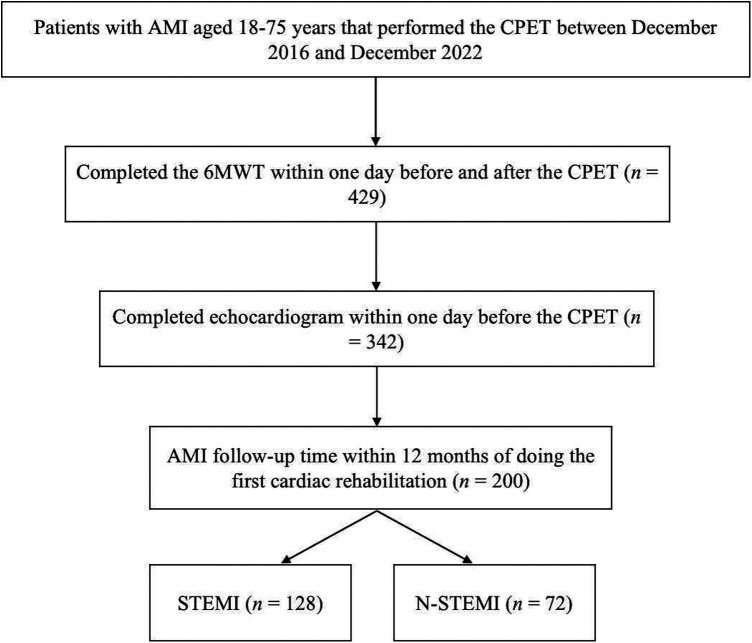
Study flowchart. AMI, acute myocardial infarction; CPET, cardiopulmonary exercise test; 6MWT, 6 min walk test; STEMI, ST-segment elevation myocardial infarction; N-STEMI, non-ST-segment elevation myocardial infarction.

A total of 467 patients with AMI, aged between 18 and 75 years, underwent CPET at the Daqing Oilfield General Hospital from December 2016 to December 2022. Of these, 429 completed the 6MWT within 24 h before or after CPET. After further refinement, 342 patients who had also undergone echocardiography within the day preceding their CPET were identified. Among them, 142 patients who had undergone AMI surgery more than 12 months earlier were excluded. Ultimately, 200 patients with AMI within the past 12 months were selected based on medical record review and ECG data. Of these, 128 had STEMI and 72 had N-STEMI (Figure 1). Their electronic medical records were analyzed to extract detailed diagnostic information, ancillary test results, body mass index (BMI), and medication history.

Table 1 shows the characteristics of these 200 AMI patients who completed the CPET, 6MWT, and ECG within 12 months of their AMI. This cohort included 188 males (93.00%), with a median age of 53 years (IQR: 46.00–61.00) and a median BMI of 25.70 kg/m^2^ (IQR: 24.22–28.40). Infarction classification included 128 patients with STEMI and 72 patients with N-STEMI.

**Table 1:** Characteristics of AMI patients.

Characteristics	AMI Group (*N* **=** 200)
Male [*n* (%)]	185 (92.50)
Age [years, *M* (P_25_, P_75_)]	52.00 (46.00, 60.75)
BMI [kg/m^2^, *M* (P_25_, P_75_)]	25.89 (24.22, 28.40)
LVEF [%, *M* (P_25_, P_75_)]	61.00 (59.00, 65.00)
History& Complications	
Hypertension [*n* (%)]	110 (54.46)
Diabetes [*n* (%)]	56 (28.00)
OMI [*n* (%)]	6 (3.00)
Drugs	
β blockers [*n* (%)]	130 (65.00)
ACEI/ARB [*n* (%)]	100 (50.00)
Antiplatelet [*n* (%)]	200 (100)
Atorvastatin [*n* (%)]	197(98.50)

AMI, acute myocardial infarction; BMI, body mass index; LVEF, left ventricular ejection fraction; OMI, old myocardial infarction; ACEI/ARB: angiotensin-converting enzyme inhibitors (ACEI) and angiotensin II receptor blockers (ARB).

### 3.2 Results of the 6MWT and CPET of subgroups

Table 2 presents the study results. A significant difference in left ventricular ejection fraction (LVEF) was observed between the STEMI and N-STEMI groups (*P* < 0.01). However, there were no significant differences between the two groups in ventilation-to-carbon dioxide output slope (VE/VCO_2_ slope), AT, or peak VO_2_ according to the CPET (*P* > 0.05). In the 6MWT results, no significant differences were found in the correlations between the 6WMD and EI_6WMD_ between the two groups (*P* > 0.05).

**Table 2:** Comparison of 6MWT and CPET between the STEMI and N-STEMI groups.

Characteristics	AMI Group	STEMI Group	N-STEMI Group	*x2**/**Z*	*P*
	(*n* = 200)	(*n* = 128)	(*n* = 72)		
Male [*n* (%)]	185 (92.50)	118 (92.19)	67 (93.06)	0.062	0.803
Age [years, *M* (*P*25, *P*75)]	52.00 (46.00, 60.75)	53.00 (46.25, 61.00)	52.00 (46.00, 58.00)	−1.062	0.288
BMI [kg/m^2^, *M* (*P*25, *P*75)]	25.89 (24.22, 28.40)	25.70 (24.22, 28.40)	25.92 (24.42, 28.31)	−0.247	0.805
LVEF [%, *M* (*P*25, *P*75)]	61.00 (59.00, 65.00)	60.00 (58.00, 64.00)	62.00 (60.00, 65.00)	−2.694	0.007**
VE/VCO_2_ slope [*M* (*P*25, *P*75)]	27.11 (23.60, 29.39)	27.11 (23.53, 29.13)	27.06 (23.79, 30.20)	−0.332	0.740
6MWD [m, *M* (*P*25, *P*75)]	416.50 (353.30, 453.00)	418.50 (348.75, 457.75)	415.50 (360.00, 450.00)	−0.205	0.838
EI_6MWD_ [km/h, *M* (*P*25, *P*75)]	4.17 (3.53, 4.53)	4.19 (3.49, 4.58)	4.16 (3.60, 4.50)	−0.213	0.832
AT [ml/kg/min, *M* (*P*25, *P*75)]	9.80 (8.20, 11.38)	9.75 (8.23, 11.58)	9.85 (7.98, 11.18.)	−0.241	0.810
EI_AT_ [(km/h), *M* (*P*25, *P*75)]	3.78 (2.82, 4.73)	3.75 (2.84, 4.85)	3.81 (2.69, 4.61)	−0.241	0.810
PeakVO_2_ [(ml/kg/min), *M* (*P*25, *P*75)]	11.85 (10.20, 14.38)	11.70 (10.20, 14.38)	12.26 (9.73, 14.38)	−0.178	0.859

*P* and *x2/Z* represent the comparison between the STEMI group and N-STEMI groups.

AMI, acute myocardial infarction; STEMI, ST-segment elevation myocardial infarction; N-STEMI, non-ST-segment elevation myocardial infarction. BMI, body mass index; LVEF, left ventricular ejection fraction; VE/VCO_2_ slope, ventilation-to-carbon dioxide output slope; 6MWD, 6 min walk distance; EI_6MWD_, exercise intensity based on 6MWD; AT, the anaerobic threshold; EI_AT_, exercise intensity based on AT; peak VO_2_, peak oxygen uptake.

** *P* < 0.01.

### 3.3 Relationship between AT and 6MWD

**Figure 2:**
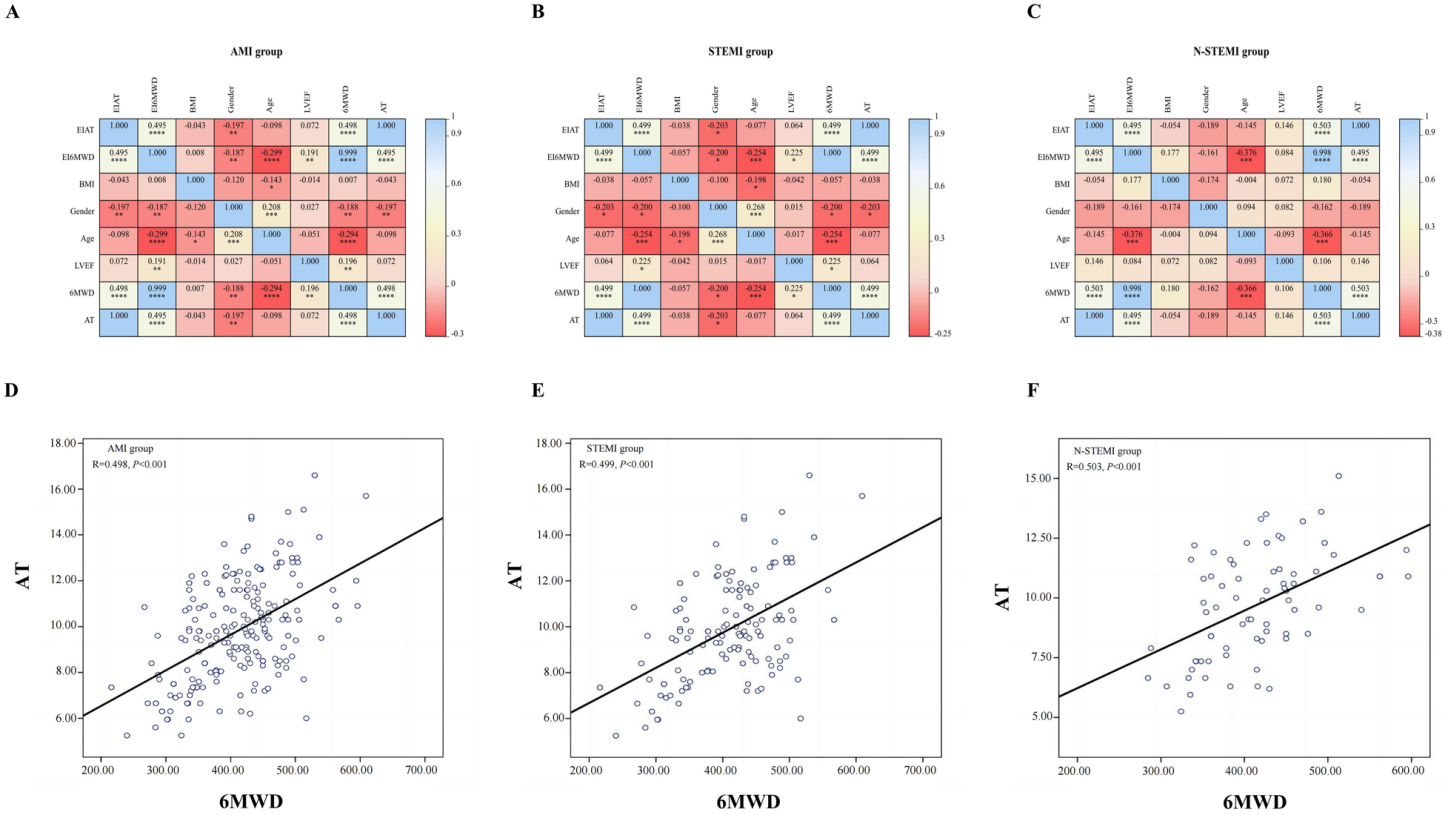
Correlation analysis results with 6MWD and AT. *, *P* < 0.05; **, *P* < 0.01; ***, *P* < 0.005; ****, *P* < 0.001; 6MWD, 6 min walk distance; AT, anaerobic threshold; AMI, acute myocardial infarction; STEMI, ST-segment elevation myocardial infarction; N-STEMI, non-ST-segment elevation myocardial infarction; **(A–C)**: the correlation between the EI_AT_, EI_6MWD_, BMI, Gender, Age, LVEF, 6MWD and AT in the three groups; **(D–F)**: the correlation between 6MWD and AT in the three groups.

Figure 2 shows the results of the univariate analyses of AT and 6MWD, as well as their correlations with other variables. A strong correlation was observed between age, AT, and 6MWD (Figure 2A–C). Additionally, a significant positive correlation was found between the AT and 6MWD in the AMI group (*r* = 0.498, *P* < 0.001) (Figure 2D). The same results were observed in the STEMI group (*r* = 0.499, *P* < 0.001) and the N-STEMI group (*r* = 0.503, *P* < 0.001).

### 3.4 Relationship between EI_6MWD_ and EI_AT_

We analyzed the AMI group and its subgroups using multiple linear regression (Table 3). The results revealed a positive correlation between EI_6MWD_ and EI_AT_ in the AMI group (*P* < 0.001). Moreover, this positive correlation was consistently observed across all subgroups in both the STEMI and N-STEMI groups (*P* < 0.001). The regression equations derived from the multiple regression analysis were as follows: for the AMI group, EI_AT_ = 0.919 × EI_6MWD_; for the STEMI group, EI_AT_ = 0.93 × EI_6MWD_; and for the N-STEMI group, EI_AT_ = 0.923 × EI_6MWD_ (Table 3).

**Table 3:** Multiple linear regression analysis results with EI_AT._

Independent variable	Regression coefficient	Standard error	Standardization regression coefficient	*t*	*P*	*F*	Adjustment *R2*
AMI group							
Constant	0.835	1.267		0.659	0.511	13.932	0.245
EI_6MWD_	0.919	0.123	0.495	7.459	<0.001****		
BMI	−0.017	0.02	−0.052	−0.831	0.407		
Gender	−0.627	0.322	−0.125	−1.95	0.053		
Age	0.009	0.009	0.068	1.028	0.305		
LVEF	−0.003	0.013	−0.017	−0.264	0.792		
STEMI group							
Constant	0.643	1.509		0.426	0.671	8.939	0.238
EI_6MWD_	0.93	0.154	0.505	6.031	<0.001****		
Gender	−0.617	0.404	−0.124	−1.527	0.129		
Age	0.011	0.012	0.083	0.982	0.328		
BMI	−0.002	0.024	−0.007	−0.084	0.933		
LVEF	−0.009	0.015	−0.047	−0.59	0.556		
N-STEMI group							
Constant	0.206	2.321		0.089	0.929	7.176	0.258
EI_6MWD_	0.923	0.198	0.491	4.667	<0.001**		
Gender	−0.778	0.543	−0.151	−1.433	0.156		
BMI	−0.074	0.044	−0.176	−1.676	0.098		
LVEF	0.039	0.031	0.13	1.257	0.213		

AMI, acute myocardial infarction; STEMI, ST-segment elevation myocardial infarction; N-STEMI, non-ST-segment elevation myocardial infarction. EI_AT_, exercise intensity based on AT; EI_6MWD_, exercise intensity based on 6MWD; BMI, body mass index; LVEF, left ventricular ejection fraction.

** *P* < 0.01.

**** *P* < 0.001.

### 3.5 Standardized regression coefficients between EI_6MWD_ and EI_AT_

The standardized regression coefficients between the EI_6MWD_ and EI_AT_ were compared using the *U*-test (Table 4). The results demonstrated that the 6MWD and AT had a moderate positive correlation in the AMI group; however, no significant differences were observed between the AMI subgroups (*P* > 0.05).

**Table 4:** Comparison of standardized regression coefficients between EI_6MWD_ and EI_AT_.

Group	*N*	*β*	*Z*	*P*
AMI group *vs.* STEMI group	200 *vs.* 128	0.495 *vs.* 0.505	−0.117	0.546
AMI group *vs.* N-STEMI group	200 *vs.* 72	0.495 *vs.* 0.491	0.038	0.485
STEMI group *vs.* N-STEMI group	128 *vs.* 72	0.505 *vs.* 0.491	0.124	0.451

*N* is the number of cases, and *β* is the normalized regression coefficient. *P* and *Z* represent the comparison relationships between each pair of groups. 6MWD, 6 min walk distance; EI_6MWD_, exercise intensity based on 6MWD; EI_AT_, exercise intensity based on AT. AMI, acute myocardial infarction; STEMI, ST-segment elevation myocardial infarction; N-STEMI, non-ST-segment elevation myocardial infarction.

## 4 Discussion

This study identified a positive correlation between EI_6MWD_ and EI_AT_ in a retrospective analysis of patients who participated in cardiac rehabilitation exercises within 12 months following AMI surgery. Previous studies have used CPET to measure exercise intensity; however, this method is often unavailable in primary hospitals due to the hight cost of the required equipment (22). In contrast, many studies investigating the relationship between 6MWD and peak VO_2_ mostly used indirect conversion methods. For instance, Linpkin (23) and Lawrence (24) utilized treadmill tests or bicycle–ergometer tests to determine cardiopulmonary function. However, the results cannot be used universally because the test platforms for the 6MWT and treadmill tests differ significantly (17). Aditionally, previous research has shown that peak VO_2_ can be estimated from 6MWD. For example, Burr et al. proposed the following equation: peak VO_2_ = 70.161 + 0.023 × 6MWD (m)—0.276 × body weight (kg)—6.79 × sex (Male = 0; Female = 1)—0.193 × resting heart rate (b.p.m.)—0.191 × age (years) (21). Therefore, the 6MWT, which is applied internationally, could be used to assess exercise capacity in cardiac patients (25, 26). Furthermore, the AT value is commonly employed as an index to quantify cardiac function (27).

Our results suggest that EI_6MWD_ and EI_AT_ are correlated in AMI patients, indicating that 91.9%–93.0% of EI_6MWD_ in AMI patients was equivalent to EI_AT_. There were no significant differences in AT, peak VO_2_, or VE/VCO_2_ slopes among the AMI subgroups at baseline (*P* > 0.05), indicating no difference in exercise tolerance or ventilation efficiency between the two groups. Furthermore, linear regression analysis demonstrated a positive correlation between 6MWD and AT to varying extents within the AMI group and its subgroups. In a further analysis, the regression equations were as follows: for the AMI group, EI_AT_ = 0.919 × EI6_MWD_; for the STEMI group, EI_AT_ = 0.93 × EI_6MWD_; and for the N-STEMI group, EI_AT_ = 0.923 × EI_6MWD_. These results demonstrated that EI_AT_ can be predicted using EI_6MWD_, suggesting that aerobic exercise intensity in AMI patients can be estimated using the 6MWT. However, compared to the New York Classification of Cardiac Function, the 6MWT, as a submaximal exercise test for cardiac rehabilitation, provides a more objective representation of patient mobility and cardiac reserve function. In addition, it has been shown to predict long-term mortality and hospitalization rates (28), a consideration widely acknowledged by international researchers. In this study, we concluded that the 6MWT can be used to measure aerobic exercise intensity in AMI patients based on the correlation between exercise intensity as assessed by 6MWT and CPET.

## 5 Limitations

This study has several limitations. Despite spanning an extended period, the sample size—particularly the number of N-STEMI subgroups—was relatively small, which may have affected the fit of the 6MWD to the AT simulation equation in the subgroup analysis. Future studies with larger patient populations are needed. Second, because blood gas and lactate levels were not measured, it remains unclear whether the 6MWT reached the AT. Future studies should incorporate these two indicators. In addition, various factors, such as walking speed and the number of trials performed may affect 6MWT results (18, 19), potentially introducing bias. Since the patients participated in the 6MWT for the first time, further standardization is necessary to improve its accuracy and reproducibility.

## 6 Conclusion

This study pioneeringly established an assessment model for anaerobic threshold equivalent exercise intensity (EI_AT_) based on the 6 min walk test distance (EI_6MWD_), filling a critical gap in exercise rehabilitation assessment for AMI patients in primary care settings. The results demonstrated that 91.9%–93.0% of AMI patients had EI_6MWD_ values equivalent to EI_AT_, indicating that the 6 min walk test effectively assesses exercise capacity and provides a foundation for personalized exercise rehabilitation plans. This assessment model is simple, cost-effective, and easy to implement in primary care settings, with the potential to significantly improve exercise rehabilitation rates and prognoses for AMI patients while promoting equitable development in cardiac rehabilitation.

Moreover, this study presents a novel solution for cardiac rehabilitation in community hospitals lacking CPET equipment, which has the potential to enhance its accessibility in developing countries and primary care settings.

However, further validation in larger populations is still needed, and the model's applicability across diverse patient groups should be explored.

## Data Availability

The raw data supporting the conclusions of this article will be made available by the corresponding authors, without undue reservation.
